# Triggering of spin-flipping-modulated exchange bias in FeCo nanoparticles by electronic excitation

**DOI:** 10.1038/srep39292

**Published:** 2016-12-19

**Authors:** Debalaya Sarker, Saswata Bhattacharya, Pankaj Srivastava, Santanu Ghosh

**Affiliations:** 1 Department of Physics, Indian Institute of Technology Delhi, Hauz Khas 110016, New Delhi, India

## Abstract

The exchange coupling between ferromagnetic (FM)-antiferromagnetic (AF) interfaces is a key element of modern spintronic devices. We here introduce a new way of triggering exchange bias (EB) in swift heavy ion (SHI) irradiated FeCo-SiO_2_ films, which is a manifestation of spin-flipping at high irradiation fluence. The elongation of FeCo nanoparticles (NPs) in SiO_2_ matrix gives rise to perpendicular magnetic anisotropy at intermediate fluence. However, a clear shift in hysteresis loop is evident at the highest fluence. This reveals the existence of an AF exchange pinning domain in the NPs, which is identified not to be oxide shell from XANES analysis. Thermal spike calculations along with first-principles based simulations under the framework of density functional theory (DFT) demonstrate that spin flipping of 3d valence electrons is responsible for formation of these AF domains inside the FM NPs. EXAFS experiments at Fe and Co K-edges further unravel that spin-flipping in highest fluence irradiated film results in reduced bond lengths. The results highlight the possibility of miniaturization of magnetic storage devices by using irradiated NPs instead of conventionally used FM-AF multilayers.

The ever-increasing demand for non-volatile memories and reading heads has led to the rapid development of anti-ferromagnetic (AF)-ferromagnetic FM) junctions, which are crucial elements of present day’s micro or nano electronics devices^1^,^2^. The interface interaction of these FM-AF layers is widely known as exchange bias (EB) effect^3^. An enhanced coercivity and asymmetric shift in the magnetic hysteresis loop are the manifestations of EB in magnetic materials. Till date, most of the EB related applicative research is focussed to different FM-AF multilayered systems only^4^,^5^,^6^. However, implementation of EB in nanoparticles (NPs) instead of hundreds of ‘nm’ thick multi-layers can provide the magnetic devices much higher order of compactness. EB effect in NPs is extensively studied for FeCo-FeCoO^7^, Ni-NiO^8^, Co-CoO^9^ and attributed to the presence of AF oxide shells surrounding FM core of the NPs. However, Ni/Co/Fe being very much prone to oxidation, it is a huge challenge to control the oxide fraction and hence the EB behaviour of these core-shell NPs. Therefore, a more robust and controlled methodology of manufacturing NPs exhibiting EB is indispensable for sleeker magnetic devices. One possibility of triggering EB in metal NPs can be partial FM to AF phase transformation in the NPs itself.

With the advent of accelerators in recent past, swift heavy ion (SHI) irradiation has emerged as a controlled tool to modify materials down to nanoscale. Both low and high energy irradiation fluences of He or, Ag ions have reportedly been used to tune the EB effect in FM-AF multi-layers^10^,^11^. Despite EB effect is observed in SHI irradiated multilayers, for SHI irradiated NPs only magnetic anisotropy (MA) effects are reported but no specific EB effects are addressed till date. However, the possibility of crystallographic or, magnetic phase transformation at very high irradiation fluence is reported^12^. Keeping these in mind, we here have utilized SHI irradiation to vary the shape/structural anisotropy of FeCo NPs with an aim to tune its MA and EB effects for magnetic device applications.

Different ions with various energies and fluences are explored in recent past to optimize the magnetic shape anisotropy for different metal NPs like Ni^13^, Co^14^, FePt^15^ etc. It should be mentioned here that presence of an insulating matrix (e.g. SiO_2_ matrix) surrounding these NPs is a prerequisite for elongating them. Also, because of their very high energy these SHIs do not alter or affect the intrinsic properties of these NPs^13^,^16^. Note that the range, nuclear stopping power, and electronic stopping power of 120 MeV Au^+9^ ions (i.e. SHIs used in our study) in SiO_2_ are respectively 15 *μ*m, 0.2 keV/nm and 14.7 keV/nm respectively as derived from SRIM 2006 software. Thus, it is very unlikely that these Au SHIs get stuck or implanted inside the ≈200 nm thicker films. We have further cross-checked from (Energy Dispersive X-ray Spectroscopy) EDX^17^ and (X-ray Photoelectron Spectroscopy) XPS measurements that there is no signature of Au in the composite thin films. Several mechanisms viz. evolution of thermal spike^16^,^18^, ion-hammering effect^19^, creep deformation^14^, shears stress induced deformation^20^ etc are described to explain SHI induced micro and macro structural changes in different materials. We noted that the shear stress in 200 MeV Iodine (I) ion irradiated Co NPs for 10^13^ ions/cm^2^ fluence is calculated only to be ≈ 0.19 GPa^21^, which is rather insufficient for producing ion-hammering effect in metal NPs. In view of this, the elongation of metal NPs (inside an insulating media) in the direction of ion beam is mostly explained by thermal spike mechanism as described in our former work^22^. Detailed thermal-pressure profiles are calculated for I irradiated Co/SiO_2_ nanocomposites and Pb irradiated SnO_2_ along with the thermal spike induced temperature distributions in refs 14 and 23 respectively. They have shown that alongside of the molten metal flow along the beam path at very high thermal spike temperatures (above 1000 K), this thermal pressure also plays a role in particle elongation.

In equiatomic FeCo alloys, magnetic to non-magnetic phase transformation is described under 35–45 GPa pressure^24^. Qiu *et al*. have also found a meta-stable AF phase for FeCo system^25^. These ideas can therefore be extended to tune the magnetic phase or structure of FeCo NPs for EB/MA studies with optimized SHI irradiation, which can give a fresh insight into the SHI-induced magnetization phenomenon. We found that the shape induced MA is predominant at intermediate fluence; while FM to AF phase transition takes over at the highest fluence where EB effect is observed.

In this article we, presumably for the first time, address triggering of the EB effect along with variation of MA in SHI irradiated NPs by state-of-the-art experimental techniques combined with first-principles-based calculations under the frame work of density functional theory (DFT).

## Experimental Section

Fe and Co foils, glued on a SiO_2_ target, were co-sputtered in a high vacuum sputtering chamber by 1.5 keV Ar fast atom beam (FAB) for depositing FeCo-SiO_2_ nanocomposite films on Si substrate. The Ar source was mounted at an angle of 45° facing towards the sputtering cathode. The substrate holder was rotated continuously for uniform deposition of the films. The relative area of the metal pieces w.r.t. the quartz plate exposed to the atom beam determines the amount of metal fraction (here 20%) in the film. The formation of FeCo alloy phase in the nanocomposite was further ensured by 2 hours of annealing in H_2_ atmosphere in a tubular furnace at 600 °C^17^. The as-grown and annealed samples were then subjected to different fluences of 120 MeV Au^+9^ SHI irradiation viz. 5.0 × 10^13^ (5e13), 7.5 × 10^13^ (7.5e13) and 1.0 × 10^14^ (1e14) ions/cm^2^ fluences at the 15UD Palletron accelerator of IUAC, New Delhi, India. Cross-sectional TEM (XTEM) samples were prepared following conventional procedure and images were recorded with FEI TITAN 80–300 microscope operating at accelerating voltage of 300 kV. Both in-plane and out-of-plane magnetic measurements were carried out in a Quantum Design MPMS SQUID magnetometer. X-ray absorption data at near and far edges (for both Fe and Co K-edges) were collected at XAFS beamline, Elettra, Italy.

## Results and Discussions

### Observation of magnetic anisotropy and exchange bias effects

**Figure 1:**
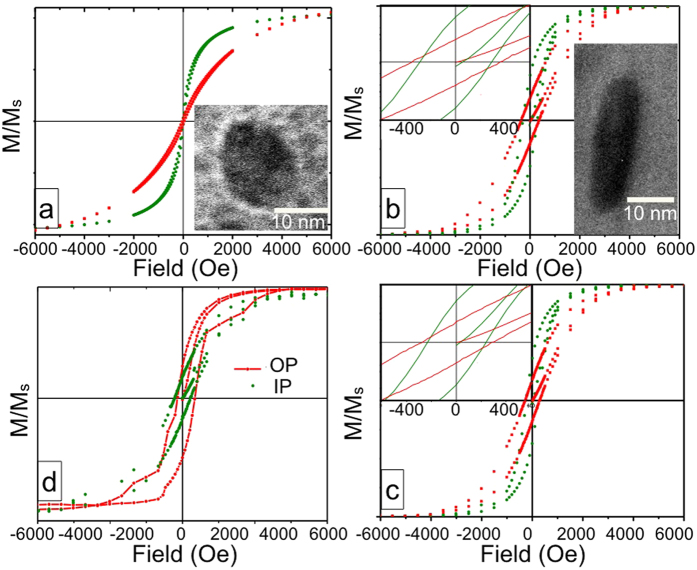
(**a**–**d**) In-plane and out-of-plane M-H plots at 5 K of unirradiated, 5e13, 7.5e13 and 1e14 films. Insets in (**a**) and (**b**) show XTEM images of the respective films. Insets of (**b**) and (**c**) show the zoomed view near the origin of M-H loops of 5e13 and 7.5e13 films respectively.

**Figure 2:**
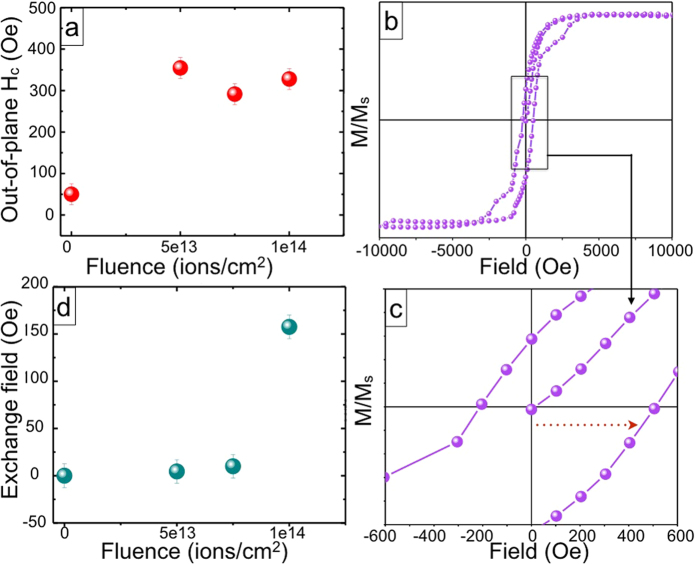
(**a**) The out-of-plane coercivity of unirradiated and irradiated films as a function of SHI fluence; (**b**) Out-of-plane M-H at 5 K and (**c**) zoomed view near the origin of the same showing exchange bias effect in 1e14 film. (**d**) Variation of exchange bias field with SHI fluence.

**Figure 3:**
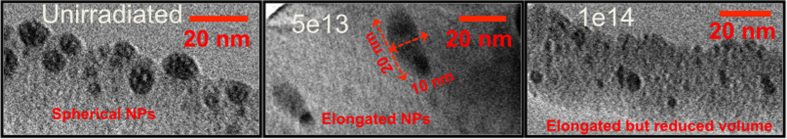
XTEM images of unirradiated, 5e13 and 1e14 films.

In Fig. 1(a–d) we have shown the in-plane and out-of-plane M-H characteristics for unirradiated and different fluence irradiated FeCo-SiO_2_ thinfilms. We noted that the out-of-plane magnetic coercivity (Fig. 2a) is increased about ≈350 times in 5e13 film as compared to the unirradiated film. Further irradiating the films at higher fluences results in reduction of out of plane magnetic coercivity. XTEM images of unirradiated and irradiated films, as shown in insets of Fig. 1(a,b), depict spherical to ellipsoid-like particle elongation in the direction of ion beam. Therefore we can say that this enhanced MA along the SHI beam direction in 5e13 film is a direct consequence of NP elongation due to the shape anisotropy introduced in the system. At higher fluences beyond 5e13, though the aspect ratio of the NPs increases, but due to fragmentation/dissolution of FeCo NPs the overall MA reduces [see Fig. 3]^17^. Next, a shift of the out-of-plane M-H loop towards positive field values is observed in 1e14 film (Fig. 2b,c), indicating the presence of an exchange bias (EB) effect. The variation of out-of-plane exchange field with SHI fluence is shown Fig. 2(d). Note that, in the in-plane M-H measurements for 1e14 film (Fig. 1(d)), we observe a slanted loop, characteristic of a magnetic hard axis. There is no EB field since the exchange coupling has been established in the perpendicular direction of the film plane. The higher out-of-plane coercivity and hence the larger out-of-plane anisotropy, is therefore responsible for the direction-dependent exchange bias in 1e14 film. We noted from our ZFC-FC data [see Fig. S1 in supporting information] that the blocking temperature rises gradually as one increases the SHI fluence and reaches ≈230 K in 1e14 film: implying systematic increase in magnetic ordering with SHI fluence. Thus this improved degree of magnetic ordering at the highest fluence (i.e. 1e14 film) has played a crucial role in triggering the EB effect. We would like to mention here that no exchange bias effect is observed from the room temperature M-H data. Note that, Chen *et al*.^26^ have reported shift in high temperature (100–400 K) M-H loops and proposed it as an EB-like phenomenon in superparamagnetic Ni NPs, having superparamagnetic blocking temperature ≈50–60 K. Keeping this possibility in mind and to avoid the occurrence of superparamagnetic phases, the M-H measurement temperature is always kept fixed to 5 K in our study that is well below the superparamagnetic blocking temperature of our all irradiated FeCo NPs [see Fig. S1].

### Understanding from electronic structure: XANES

**Figure 4:**
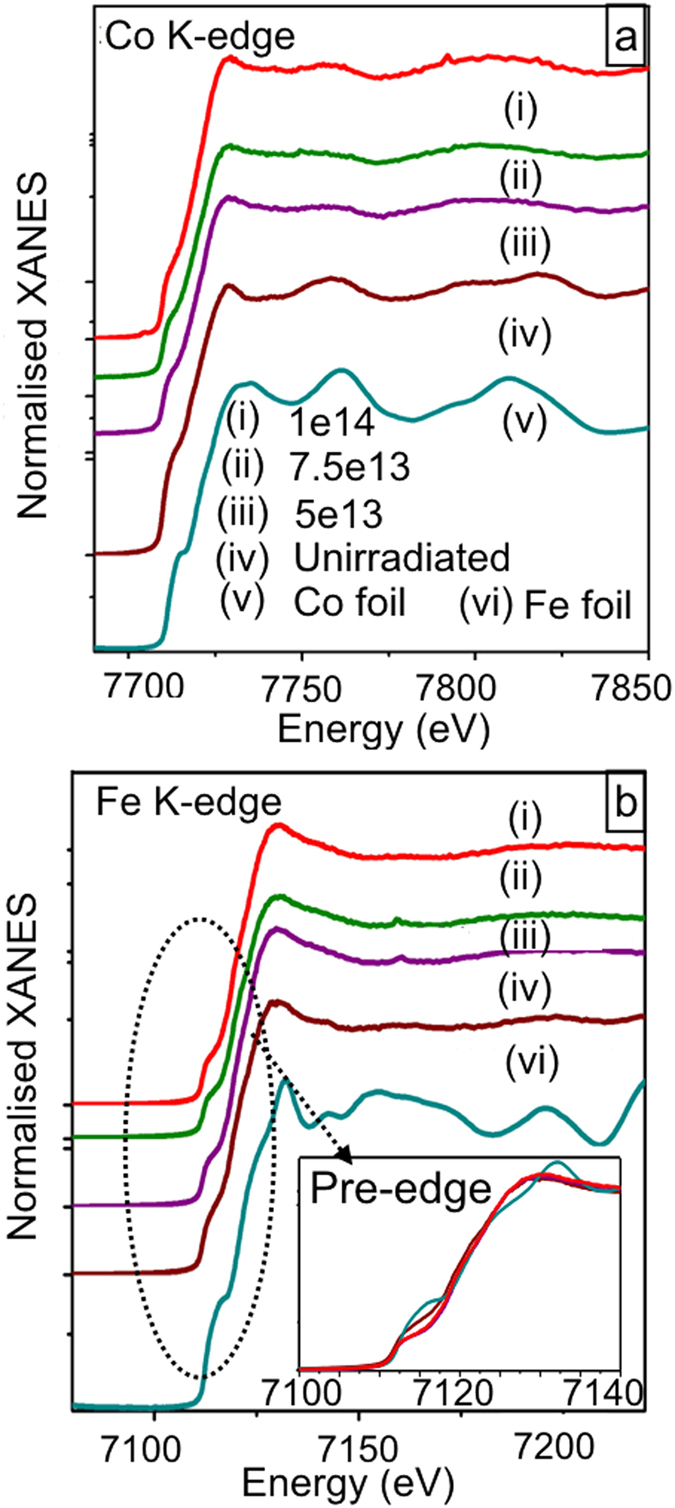
XANES at (**a**) Co and (**b**) Fe K-edges showing pre-edge feature in all irradiated and unirradiated films similar to the respective metal foils. Inset (**b**) shows a close up of the pre-edge at Fe K-edge.

Occurrence of AF FeCo phase has been reported before by Qiu *et al*.^25^. Thus, the interaction of AF domains with FM domains can be a probable reason for this anomalous triggering of EB effect in irradiated FeCo NPs in 1e14 film. One of the possibilities is formation of AF oxides at the shell of the elongated NPs. To cross-check this, we have performed X-ray absorption near edge spectroscopy (XANES) [see Fig. 4(a,b)] measurements at both Co and Fe K-edges. Note that a prominent pre-edge feature is present in unirradiated and in all irradiated films, which are similar to that of the corresponding metal foils. The presence of these pre-edge peaks, representing 1s to 3d transitions, clarifies that the NPs predominantly sustain T_*d*_ symmetry like metals and not O_*h*_ symmetry as oxides. However, having a closer look at the pre-edge (inset Fig. 4b) at Fe K-edge, we find that there is an edge shift in all irraidated films confirming partial oxidation in post-irradiated FeCo NPs. But, the overlap of 5e13 and 1e14 XANES spectra ascertains that oxide content is more or less same in both the films. Thus formation of AF oxides can not be a viable reason for the observed EB effect only in 1e14 film. Moreover, our core level photo-emission data (not shown) has ruled out the possibility of silicide formation in the 1e14 film. Also, no signature of oxide shell formation is evident from XTEM images.

### Understanding from electronic structure: DFT and thermal spike modelling

To understand the reason behind this emerging EB effect only at the highest fluence and how the 3d electrons are playing a role in it, we needed to have a look at the electronic structure of the systems. First we have calculated the lattice temperature (*T*_*l*_)^18^ and pressure (*P*)^23^ profiles in films irradiated at different fluences using thermal spike model. The electronic (*T*_*e*_) and lattice (*T*_*l*_) temperatures are found by solving the set of partial differential equations:


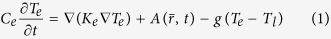



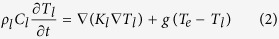


*C*_*e*_, *C*_*l*_, *K*_*e*_, *K*_*l*_ are the specific heats and thermal conductivities of electronic and lattice sub-systems respectively. *ρ*_*l*_ is the material density. 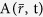 is the energy transferred to the electrons from heavy ion at a time t and at a distance r from the ion’s path. 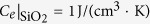 and 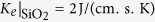; we adapted the lattice specific heat and thermal conductivity values of metals and SiO_2_ from the works of Wang *et al*.^27^ and Kumar *et al*.^13^. The expression for 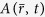 is taken as described by Meftah *et al*.^18^. More details are described in ref. 22. Thereafter, the thermal pressure profile *P(r, t*) is calculated in order to understand the pressure effect into the system. Note that the increase in pressure is


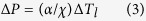


where, *α, χ* are the volume expansion co-efficient and adiabatic compressibility of FeCo respectively and Δ*T*_*l*_ is the increase in lattice temperature. Therefore,


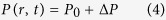


gives us the thermal pressure profile of the system.

**Figure 5:**
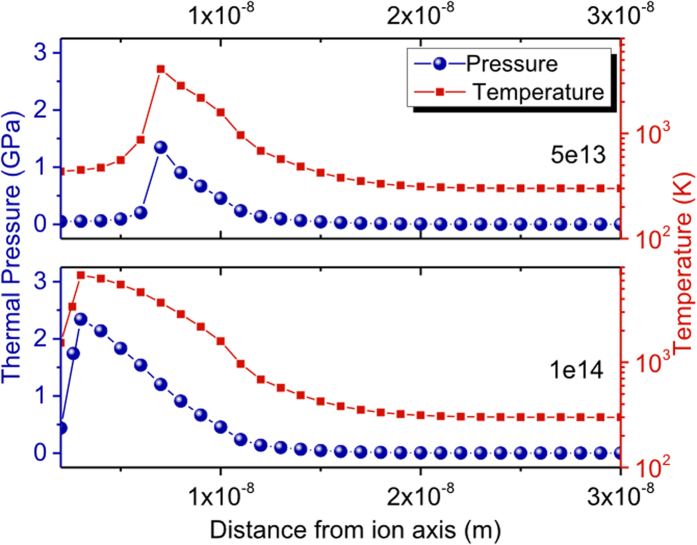
Thermal spike model derived lattice temperature and pressure profiles (**a**) 5e13 and (**b**) 1e14 films.

To calculate the lattice temperature profiles, the statistically averaged minor dimensions of the NPs in 5e13 and 1e14 films are taken as 6 and 3 nm respectively from grazing incident small angle X-ray scattering measurement^22^. Upon solving Equations 1 and 2 we find the lattice temperature goes upto 4000 K in 5e13 and 6000 K in 1e14 film (see Fig. 5). From our calculated *P(r, t*) profiles as in Fig. 5, we observe thermal pressure rises up to 1.31 and 2.02 GPa in 5e13 and 1e14 films respectively. It’s been reported that Fe_0.5_Co_0.5_ undergoes a pressure-induced bcc FM to hcp non-FM phase transition for an applied pressure of 30–40 GPa^24^. Based on this observation, we conclude that the thermal pressure developed in our system is not sufficient enough for magnetic phase transformation. Therefore, origin of the observed EB effect requires more fundamental understanding at the atomistic label. The formation of stable bcc FeCo phase in our unirradiated NPs was confirmed from detailed extended X-ray absorption fine structure (EXAFS) analysis^17^. Keeping this in mind, we started our calculations, with a bcc FeCo cluster (viz. (FeCo)_8_). We have also confirmed that this structure is the global minimum structure at that size using cascade genetic algorithm implementation^28^,^29^. Note that Wu *et al*. have shown that the binding energy/cell (−0.51 eV/cell) is minimum for (FeCo)_8_ cluster when it is in bcc configuration^30^. Following the thermal spike temperatures raised in respective fluences, we have performed *ab initio* molecular dynamics (MD) simulations under the frame work of DFT to first understand the structural changes of the bcc (FeCo)_8_ cluster at those temperatures (i.e. fluences). For this we have used all electron based FHI-aims code, which uses numerical atom centered basis set^31^. The exchange and correlation functional is taken from generalized gradient approximations (GGA) as in PBE^32^ implementation which is duly validated with more advanced HSE06^33^ hybrid functionals. To draw analogy with our annealing conditions, we have chosen 800 K for the MD run of the (FeCo)_8_ cluster corresponding to the unirradiated film. We then raised the temperature up to 4000 and 6000 K as per our thermal spike model findings for 5e13 and 1e14 films respectively. At each temperature the system was kept for 8 ps MD using Nose-Hoover thermostat and following that it was cooled to room temperature [T = 300 K] via 4 ps MD simulation. Thus, a fast quenching was introduced in our model system to see the structural deformation when the FeCo NPs are irradiated under different fluences. Following this the structure is optimized to its nearest minima. Finally to investigate the directional magnetism of these structures, we have performed non-collinear magnetic calculations using Vienna *ab initio* simulation package^34^,^35^ with Projector-augmented wave (PAW) pseudopotential. There is very minimal (less than 0.001 Å in bond length) structural changes in the fully relaxed optimized structures using plane-wave based VASP and all-electron based FHI-aims.

**Figure 6:**
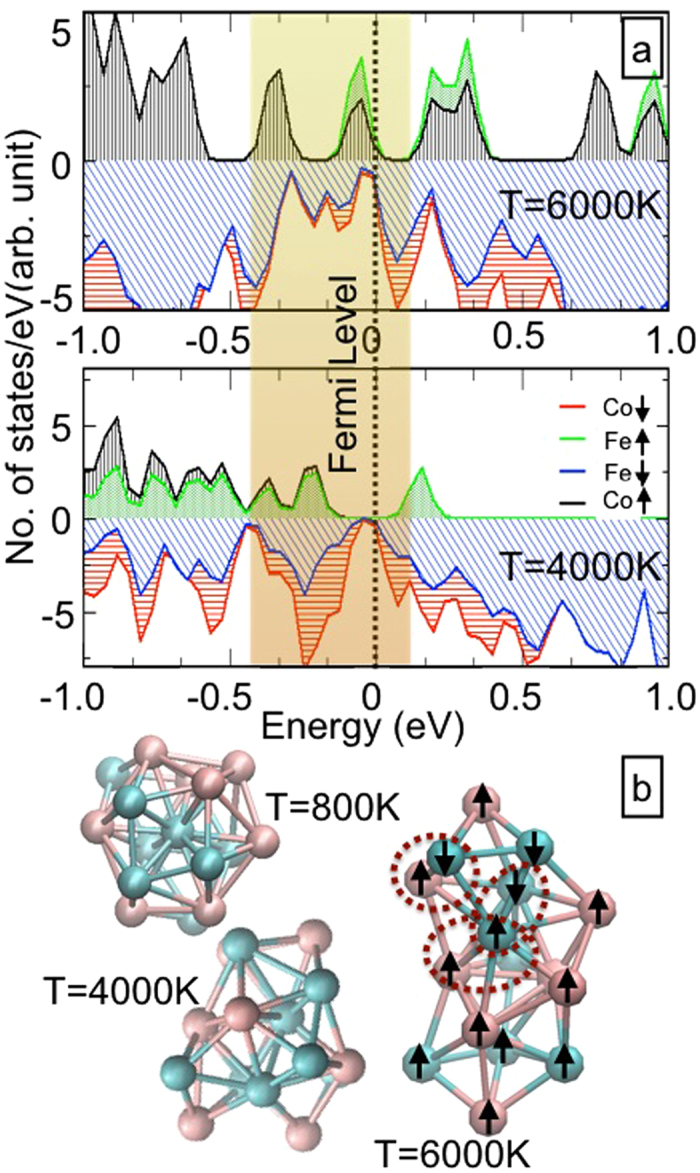
(**a**) Atom-wise DOS near Fermi edge for T = 4000 K and T = 6000 K structures. (**b**) The structures at the T = 800 K, T = 4000 K and T = 6000 K are shown. For the structure at T = 6000 K, the spin alignment of the respective atoms are shown by arrows when the magnetic field is applied along y-direction.

**Figure 7:**
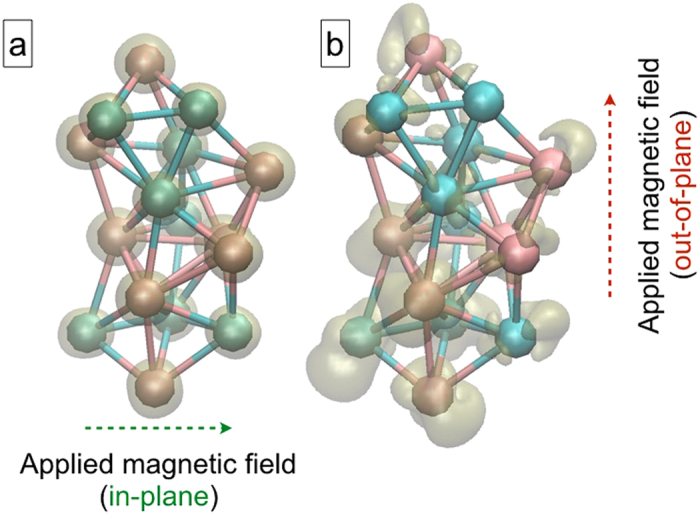
The spin-density distribution is shown for T = 6000 K structure when the magnetic field is applied (**a**) along x-direction (in-plane) and (**b**) along y-direction (out-of-plane) in our non-collinear calculations.

The atom-wise spin-Density of states (DOS) is plotted (near the Fermi energy) in Fig. 6a for the DFT structures corresponding to 5e13 and 1e14 films respectively (see Fig. 6b). This shows that parallel orientation of atom-spins (of a few Co atoms) in T = 4000 K structure (i.e. 5e13 film) is flipped in T = 6000 K structure (i.e. 1e14 film). Further, we noted from the total DOS and experimental XPS valence band data in ref. 22 that the density of valence electrons is maximum for 5e13 film: indicating more polarization of 3d electrons therein. It’s therefore inferred that this is the reason behind higher MA in the T = 4000 K structure (5e13 film). The spin flipping in the T = 6000 K structure is more prominent from the spin-moment plot (see Fig. 6b), where the hirshfeld spin moments of all individual atoms are shown when the magnetic field is applied along y-dir (out-of-plane). This not only reduces the total magnetization but also form few AF domains within the FeCo cluster. We therefore conclude that the interaction between these AF and FM domains is responsible for the observed EB in 1e14 film. In order to establish that the directional nature of EB is a direct consequence of strong perpendicular anisotropy in the FeCo NPs, we have plotted the variation of the spin-density distribution under the applied magnetic field both in x-direction and y-direction of the T = 6000 K structure (see Fig. 7). It’s clearly evident that strong perpendicular anisotropy in the FeCo NPs results the out-of-plane distorted nature of the electronic spin density. The later validates the reason behind the directional nature of the observed EB effect.

### Validation from local atomic structure: EXAFS

**Figure 8:**
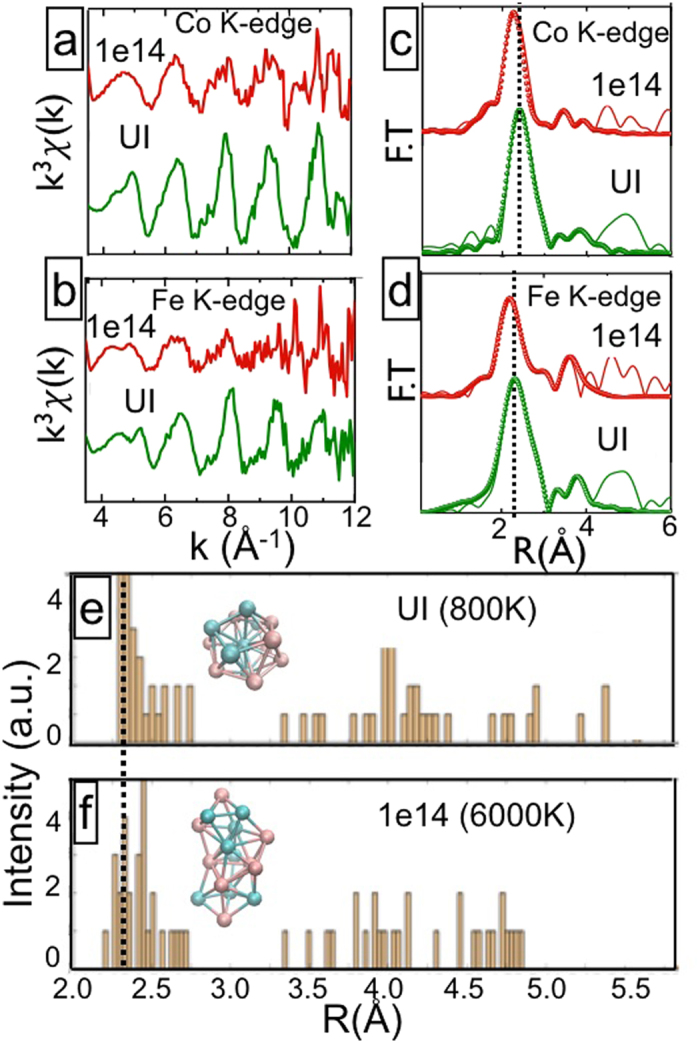
k^3^ weighted EXAFS (**a**,**b**) and the corresponding FT’s (**c**,**d**) spectra at Co and Fe K-edges for unirradiated (UI) and 1e14 films; (**e**,**f**) show the pair correlation function plots of the clusters at T = 800 K (**e**) and T = 6000 K (**f**): both showing reduction in bond length at higher temperature (i.e. fluence).

To validate our theoretical conclusion, we have further analyzed the local atomic environment with EXAFS. The k^3^ weighted EXAFS at both Co and Fe K-edges and the corresponding Fourier transform (FT) spectra are presented in Fig. 8(a–d). This clearly shows that the first nearest neighbour bond length (BL) gets reduced as one goes from unirradiated (UI) to 1e14 film. Because of high fluence (i.e. temperature) and disorder, the BL in principle, should increase in 1e14 film. This off-the-trend reduction in BL can’t be justified on the basis of partial oxidation. One of the possible reasons for this is spin-flipping. The attraction between the anti-parallel spins dominates the atomic arrangement and reduces the nearest neighbour BL. Note that the pair-correlation function (see Fig. 8e,f) of our respective MD structures also show the similar trend of BL reduction as our EXAFS FT findings: thus justifying our explanation about the emergence of EB effect at the highest fluence. Note that, our observation of presence of AF domains in T = 6000 K cluster is also checked and validated in model clusters including oxygen atoms as well.

## Conclusion

In conclusion, we have tailored the magnetic shape anisotropy and have triggered a directional EB effect in FeCo NPs by monitoring SHI fluences for magnetic device applications. The MA first increases in 5e13 film due to NP elongation along SHI beam direction and improved polarization of 3d electrons. The out-of-plane coercivity then reduces due to fragmentation and dissolution of NPs beyond 7.5e13. The observed EB effect in the highest fluence irradiated film is explained by formation of AF domains as a consequence of spin-flipping at higher thermal spike temperature.

## Additional Information

**How to cite this article**: Sarker, D. *et al*. Triggering of spin-flipping-modulated exchange bias in FeCo nanoparticles by electronic excitation. *Sci. Rep.* **6**, 39292; doi: 10.1038/srep39292 (2016).

**Publisher's note:** Springer Nature remains neutral with regard to jurisdictional claims in published maps and institutional affiliations.

## Supplementary Material

Supplementary Information
