# Supplementary material for: Triggering of spin-flipping-modulated exchange bias in FeCo nanoparticles by electronic excitation
Source: Sci Rep. 2016 Dec 19;6:39292. doi: 10.1038/srep39292 (PMC5171942; doi:10.1038/srep39292)
Supplement: Supplementary Information [file srep39292-s1.pdf]

Triggering of spin-flipping-modulated exchange bias in FeCo nanoparticles by electronic excitation

Debalaya Sarker*, Saswata Bhattacharya+, Pankaj Srivastava+, and Santanu Ghosh*

[1]Department of Physics, Indian Institute of Technology Delhi, Hauz Khas 110016, New Delhi, India

*debalaya.sarker@physics.iitd.ac.in (DS), santanu1@physics.iitd.ac.in (SG)

Supporting Information:

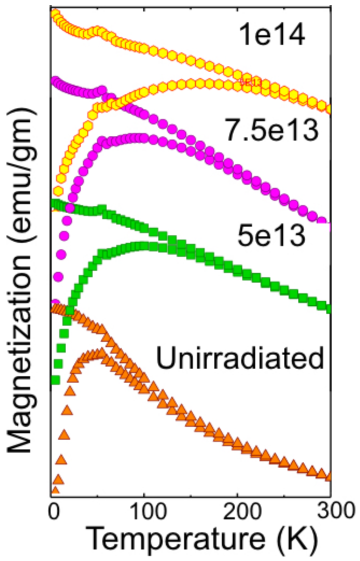

Figure S1: ZFC-FC plots for unirradiated and different irradiated films showing gradual increase in blocking temperature with irradiation fluence.

The gradual increase in blocking temperature with increasing fluence is observed from figure S1. Thus this improved degree of magnetic ordering at the highest fluence (i.e 1e14 film) is evident that has played a crucial role in triggering the EB effect in the same.
